# Learning in peer teaching of patient relations and communication skills at the “Anamnesegruppen” Munich – proof-of-concept and lessons learned

**DOI:** 10.3205/zma001400

**Published:** 2021-01-28

**Authors:** Raphael Kunisch, Peter Zimmermann, Natalie Berges, Malte Nitzschke, Felix Schweiger, Mira Seidl, Marc Weidenbusch

**Affiliations:** 1 Friedrich-Alexander-Universität Erlangen-Nürnberg, Allgemeinmedizinisches Institut, Universitätsklinikum Erlangen, Erlangen, Germany; 2 Technische Universität München, Fakultät für Medizin, Munich, Germany; 3 Ludwig-Maximilians-Universität München, Medizinische Fakultät, Munich, Germany; 4 Ludwig-Maximilians-Universität München, Psychologische Fakultät, Munich, Germany; 5 Ludwig-Maximilians-Universität München, Nephrologisches Zentrum, Medizinische Klinik und Poliklinik IV, Klinikum der LMU München, Munich, Germany

**Keywords:** anamnesis groups, digital teaching, e-learning, relationship medicine, patient interview, Balint, medical history, student teaching, peer-teaching, corona, COVID-19, pandemic

## Abstract

**Background: **Due to the ban on classroom teaching during the pandemic, the Munich “Anamnesegruppen” had to be switched to e-learning at short notice. There were no established concepts for this, which is why digitalization was piloted and evaluated for feasibility.

**Student “Anamnesegruppen”: **“Anamnesegruppen” have existed for over 50 years and are organized as independent student peer teaching. In small groups of medical and psychology students, interviews with patients are conducted once a week during the semester. This is followed by a feedback and discussion round, in which ethical and professional questions are discussed in addition to the patient's medical history. The goal is to train the participants' ability to communicate and reflect.

**Adaptation to digital methods: **The anamnesis seminars have been moved to a virtual group room using video conference. Patients were mainly recruited from the participants' circle of acquaintances. The group size was set at eight people each in four groups and supervised by a pair of student tutors. Confidentiality and data protection declarations were obtained in writing.

**Results:** By switching to digital anamnesis groups, all four groups were successfully completed. Both the final supervision of the tutors and the electronic evaluation of the participants yielded positive feedback. Compared to the two previous evaluations of the semesters in classroom sessions, there were no significant differences in the evaluation.

**Discussion: **The continuously good evaluation results, which did not differ between the digital format and the classroom course of the previous semesters, show that an ad hoc conversion to digital teaching is possible. We want to stress the fact that elements reflecting the doctor-patient relationship were successfully preserved. For the similarly structured Balint groups, virtual sessions may also be considered. Further research, especially prospective, is desirable in order to better understand the possibilities of digital teaching in this area.

## Zusammenfassung

**Hintergrund: **Durch das Verbot von Präsenzunterricht im Rahmen der Pandemie mussten die Münchner Anamnesegruppen kurzfristig auf E-Learning umgestellt werden. Hierfür lagen keine etablierten Konzepte vor, weshalb eine Digitalisierung pilotiert und auf Machbarkeit evaluiert wurde.

**Studentische Anamnesegruppen: **Anamnesegruppen existieren seit über 50 Jahren und werden in eigenständigem studentischen Peer Teaching durchgeführt. In Kleingruppen aus Medizin- und Psychologiestudierenden werden einmal wöchentlich während des Semesters Gespräche mit PatientInnen durchgeführt. Hierauf folgen eine Feedback- sowie Diskussionsrunde, in der neben der Krankengeschichte auch ethische und professionelle Fragen erörtert werden. Ziel ist es die Kommunikations- und Reflexionsfähigkeit der Teilnehmenden zu schulen.

**Adaptation auf digitale Methoden: **Die Anamneseseminare wurden in einen virtuellen Gruppenraum mittels Videochat verlagert. PatientInnen wurden überwiegend aus dem Bekanntenkreis der Teilnehmenden rekrutiert. Die Gruppengröße wurde auf jeweils acht Personen in vier Gruppen festgelegt und durch ein Paar von studentischen TutorInnen betreut. Schweigepflichts- und Datenschutzerklärungen wurden schriftlich eingeholt.

**Ergebnisse: **Durch die Umstellung auf digitale Anamnesegruppen konnten alle vier Gruppen erfolgreich durchgeführt werden. Sowohl in der Abschlusssupervision der TutorInnen als auch in der elektronischen Evaluation der Teilnehmenden wurde die Veranstaltung positiv bewertet. Im Vergleich zu den zwei vorausgegangenen Evaluationen der Semester in Präsenzveranstaltung zeigten sich keine signifikanten Unterschiede in der Bewertung.

**Diskussion: **Die unverändert guten Evaluationsergebnisse, welche sich in der digitalen Durchführung nicht von der Präsenzveranstaltung der letzten Semester unterschieden, zeigen, dass eine ad-hoc Umstellung auf digitale Lehre möglich ist. Dabei scheint uns insbesondere interessant, dass auch Elemente zur Reflektion der Arzt-Patient-Beziehung erfolgreich erhalten werden konnten. Für die ähnlich konzipierten Balintgruppen lässt sich möglicherweise auch eine elektronische Durchführung andenken. Weitere, insbesondere prospektive, Forschung ist wünschenswert, um die Möglichkeiten der digitalen Lehre in diesem Bereich besser kennen zu lernen.

## Background

Due to the SARS-CoV-2 pandemic, the state government has banned classroom instruction at Bavarian universities [https://www.gesetze-bayern.de/Content/Document/BayIfSMV_7] The tutors (TUT) of the independent student peer teaching project “Anamnesegruppen” therefore decided to digitize the event in the summer semester 2020.

## Student anamnesis groups (ANAG)

ANAGs go back to Wolfram Schüffel, who worked together with Thure von Uexküll in Ulm in 1969. Since 1976 ANAGs were carried out as student peer teaching and within the next decade they spread throughout Germany and Austria through student initiative. It is estimated that about 5% of the medical students in Germany go through the mostly voluntary course. ANAGs are mostly organized interprofessionally by students of medicine and psychology [1].

**Figure 1:**
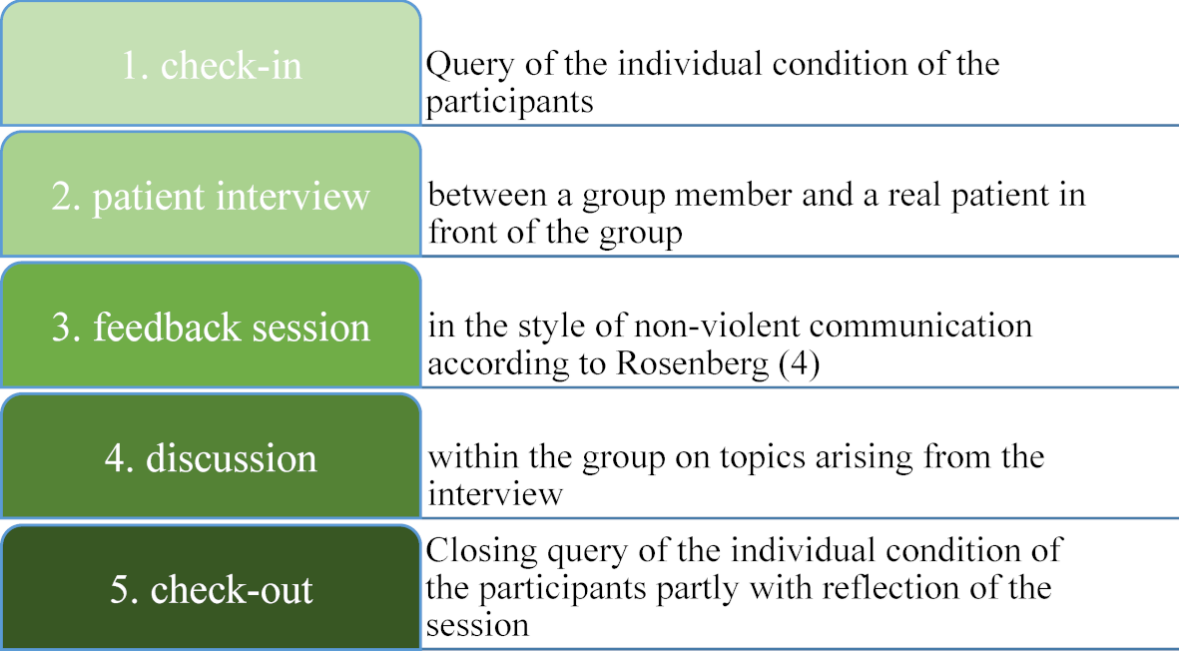
Structure of ANAGs

**Figure 2:**
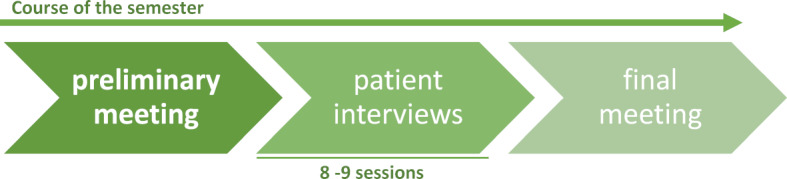
Course of the semester

The content of the seminar is the name-giving patient interview (in German: “Anamnese”) as well as the resulting relationship and its ethical and professional aspects; similar to the Balint groups, which serve the reflection of the doctor-patient relationship and have been a compulsory part of several specialist training courses in Germany for over 40 years [2]. Attending the ANAG, participants learn and improve patient contact already as a student, which becomes crucial later on in professional life. Therefore a bio-psycho-social understanding of illness and a holistic view of the person are essential. Instead of factual knowledge, the focus is on process-oriented learning in the peer group and the emotional experience of communication [3]. In addition to the patient interview, there are structured elements that are intended to positively influence the group dynamics. The structure of the ANAGs sessions is shown in figure 1 (Fig. 1). ANAGs are offered in Munich as a compulsory elective but can also be participated in as a non-compulsory course. Each ANAG consists of ten participants and two student TUTs, which are as heterogeneous as possible in terms of subject, gender and experience. The responsible TUTs are supervised by trained doctors and psychologists. During the semester there are evening meetings once a week, on the same day of the week at the same time, lasting about 2.5 hours (3 semester hours per week) in changing departments of the Munich university clinics (see figure 2 (Fig. 2)).

Prerequisite for participation is regular attendance and participation with a maximum of two missed sessions – also for those not taking the course as an elective subject. The TUTs ensure that these rules are obeyed. Participants who take the course as elective have to write a reflection report which is evaluated together their participation by the TUTs additionally to their participation (see attachment 1 ). Further information about structure and procedure of the ANAGs can be found in a separate publication [3].

## Digitalization

In order to carry out the ANAGs despite the pandemic’s restrictions, the TUTs decided together to switch to digital anamnesis groups (dANAGs). When selecting the platform, confidentiality was prioritized, and the end-to-end encrypted video conferencing software “Cisco Webex” was chosen. TUTs and participants were in the virtual web conference room simultaneously for the duration of the dANAG. Duration, content, and time of the appointments were left unchanged compared to classroom teaching. Patients were invited to the conference room via an e-mail link once and only for the duration of the anamnesis interview. The group size was limited to a maximum of eight participants. The students were additionally obliged to recruit a patient by their participation and received a guideline (see attachment 2 ). Supported by some physicians and psychologists, a back-up pool of patients was set up. A total of eight TUTs were organizing the dANAGs, two of whom designed the online format. In addition, a supervisor and 25 different patients were involved.

All participating students signed a confidentiality agreement. Participating patients received a declaration of consent and privacy, which also served as a detailed written explanation and was sent back to the TUTs. 

## Results

29 students registered and were distributed among four dANAGs. Except for short interruptions of the transmission there were no disturbances and all dANAGs could be completed. At the end of the semester a final evaluation was conducted (response rate 69%). Since the ANAGs obtained the accreditation as a compulsory elective subject of the medical faculty, the evaluation survey was carried out in the seven preceding semesters. Initially it was obtained written on paper and since the previous semester collected electronically via [https://www.soscisurvey.de/] with identical content. The course was described just as successful in all dANAGs. The reflection of the doctor-patient relationship, as well as ethical questions, were assessed by the experienced TUTs as comparable to the presence teaching. This is also supported by the fact that the comparison of the final evaluations of the dANAGs and ANAGs of the previous semester shows no differences (see attachment 3 ). The participants’ free-text answers regarding ANAG and dANAG cover the same categories in terms of content (see table 1 (Tab. 1)), with feedback and group discussion as aspects most often mentioned.

**Figure 3:**
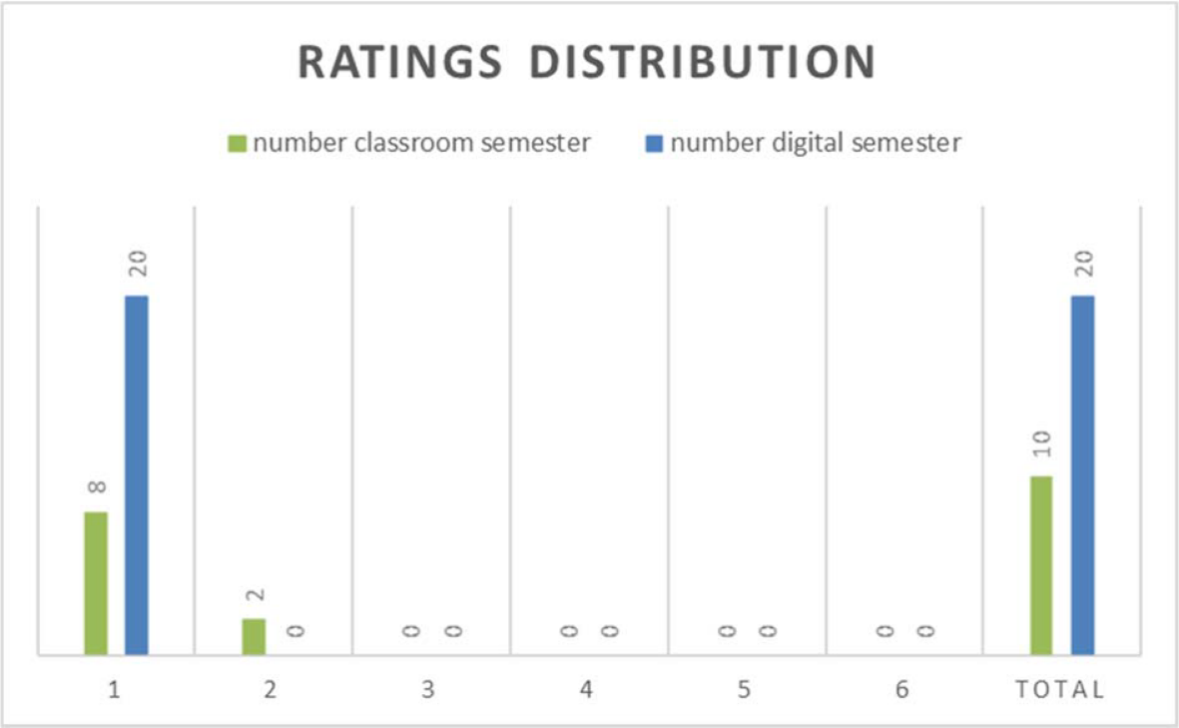
Ratings distribution of ANAGs

The grading of the elective participants did not differ between the presence and digital format, but there was a doubling of the number of participants who took the ANAGs as a compulsory elective (see figure 3 (Fig. 3)).

At the end of the semester, the TUTs emphasized that the conversations had shown themselves to be comparable to presence on both the verbal and non-verbal levels. Due to the more intimate setting of the home environment, there was even more openness reported in some cases. Structural elements of the ANAGs could be transferred into the digital world and were mentioned as factors for successful reflection and group dynamics (games to get to know each other, feedback, check-in and discussion) (see figure 1 (Fig. 1)).

## Discussion

Analyzing the evaluation, dANAG was well received. We consider the doubling of the number of participants who took the course as compulsory elective as an indication that the reduction in classroom teaching led to a greater demand for digital compulsory electives. It is important that dANAGs are accompanied by experienced TUTs who specifically reflect the group dynamics and structure the dANAGs. The digitalization is transferable to other locations where ANAGs are already established since the basic principle is similarly organized nationwide and usually exists independently of the local university curriculum. Also, the similarly structured Balint groups [2] could be digitized under special circumstances, e.g. if classroom teaching is not possible due to contact restrictions but also if participants are spread across a wide distance. A randomized and controlled investigation of the effects of dANAGs could be the next research step.

## Acknowledgement

The authors would like to thank the student tutors and student participants for their support in this work.

## Authorship

RK and PZ share the first authorship and were responsible for writing the manuscript as well as collection and evaluation of the data. NB and MN were involved in the development of the manuscript. FS and MS developed and conducted the evaluation. FS and MW revised and proof-read the manuscript and were involved in the data evaluation.

## Competing interests

The authors declare that they have no competing interests. 

## Supplementary Material

Evaluation sheet – summer semester 2020

A short guide to patient recruitment for the virtual anamnesis group

Results of the semester evaluations of winter semester 2019/20 and summer semester 2020

## Figures and Tables

**Table 1:**
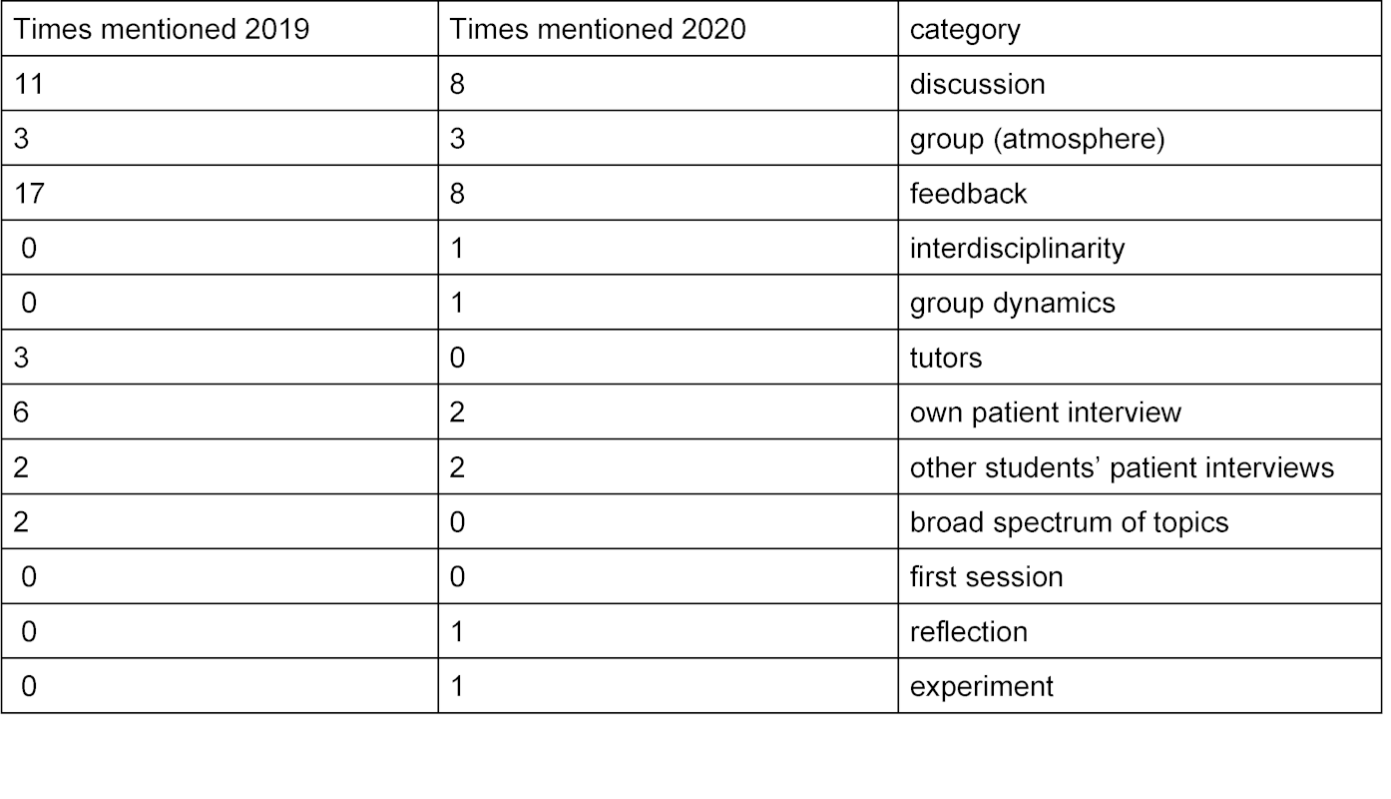
Categories of replies in the free-text field: “What was most beneficial for you resp. what did you like most?” Total response summer term 2019: 21; summer term 2020: 20
