# Supplementary material for: Learning in peer teaching of patient relations and communication skills at the “Anamnesegruppen” Munich – proof-of-concept and lessons learned
Source: GMS J Med Educ. 2021 Jan 28;38(1):Doc4. doi: 10.3205/zma001400 (PMC7899106; doi:10.3205/zma001400)
Supplement: Evaluation sheet – summer semester 2020 [file JME-38-1-4-s-001.pdf]

Attachment 1: Evaluation sheet - summer semester 2020.

Medical history groups of LMU and TU Munich – summer semester 2020

tutors: ______________________

participant: ______________________

matriculation number: ____________________________

Grading for medical elective students

The grading is based on a point system in which a maximum score of 100 can be achieved. There is a basic area in which the requirements **must** be fulfilled (conversation and presence) and another area in which the participant **can** fulfill the requirements. The corresponding grading results as follows:

Basics:

- ☐ Conversation with a patient conducted in front of the group & presence fulfilled (not more than 2 days of absence)
  = 60 points

Reflection

- ☐ Reflection report submitted (contains 600 words - a deviation of +/- 10% is tolerated)
  = 0 - 2,5 points
- ☐ Formal criteria are adequately fulfilled (spelling, expression, form, ...)
  = 0 - 2,5 points
- ☐ Reflection report contains a clear discussion of the topic (about one's own patient interview and/or about oneself in regard to the group and/or about personal development)
  = 0 - 15 points

Participation:

- ☐ The participant has regularly participated actively
  = 0 – 5 points
- ☐ The contributions were constructive for the interview/ the discussion/ the dynamics of the group (e.g.: initiates new discussion topics, observes and articulates the group events, shows development, gives constructive feedback)
  = 0 - 15 points

The points are added up to form a percentage:

90 - 100 = very good

80 - 89,5 = good

70 - 79.5 = satisfactory

60 - 69,5 = sufficient

< 60 = insufficient (failed)

Attachment 1 to: Kunisch R, Zimmermann P, Berges N, Nitschke M, Schweiger F, Seidl M, Weidenbusch M. *E-learning in peer-teaching of patient relations and communication skills at the "Anamnesegruppen" Munich – proof-of-concept and lessons learned.* GMS J Med Educ. 2021;38(1):Doc4. DOI: 10.3205/zma001400
