# Supplementary material for: Learning in peer teaching of patient relations and communication skills at the “Anamnesegruppen” Munich – proof-of-concept and lessons learned
Source: GMS J Med Educ. 2021 Jan 28;38(1):Doc4. doi: 10.3205/zma001400 (PMC7899106; doi:10.3205/zma001400)
Supplement: A short guide to patient recruitment for the virtual anamnesis group [file JME-38-1-4-s-002.pdf]

Attachment 2: A short guide to patient recruitment for the virtual anamnesis group

Let us briefly reflect on the core idea of our style of patient interview:

To grasp a person’s psycho-social state as well as his personal structure which may be challenged by illness. In doing so, we will also pay special attention to the secondary and tertiary consequences of disease with effects on all areas of life (job, family, friends, ...) as well as our own concept of health and disease.

A suitable candidate for a virtual patient interview therefore ideally is a person who, on the one hand, has been affected in one or more of the above-mentioned areas by a disease that has already existed for some time and/or is only acutely occurring. The type or severity of the disease only partially plays a role – what matters are the individually perceived changes of the person affected and their willingness to talk about them.

Attachment 2 to: Kunisch R, Zimmermann P, Berges N, Nitschke M, Schweiger F, Seidl M, Weidenbusch M. *E-learning in peer-teaching of patient relations and communication skills at the “Anamnesegruppen” Munich – proof-of-concept and lessons learned.* GMS J Med Educ. 2021;38(1):Doc4. DOI: 10.3205/zma001400
