# Supplementary material for: Learning in peer teaching of patient relations and communication skills at the “Anamnesegruppen” Munich – proof-of-concept and lessons learned
Source: GMS J Med Educ. 2021 Jan 28;38(1):Doc4. doi: 10.3205/zma001400 (PMC7899106; doi:10.3205/zma001400)
Supplement: Results of the semester evaluations of winter semester 2019/20 and summer semester 2020 [file JME-38-1-4-s-003.pdf]

Attachment 3: Results of the semester evaluations of winter semester 2019/20 and summer semester 2020**.**

The mean values of the answers rounded to one decimal. Broken down into subgroups: Medical students, psychology students and participants of the elective subject.

Five-level Likert scale: 1=completely disagree, 2=Mostly disagree, 3=Neutral, 4=Mostly agree, 5=Completely agree.

	winter semester 2019/20 - classroom teaching				*summer semester 2020 - digital teaching*			
Evaluation question	***total***	*med.*	*psych.*	*elective*	***total***	*med.*	*psych.*	*elective*
1. there was a pleasant group atmosphere in my group	4.8	4.9	4.8	4.7	5.0	4.8	5.0	5.0
2. the group size was pleasant	4.7	4.7	4.8	4.9	5.0	5.0	5.0	5.0
3. the participation in the anamnesis group motivated me to think for myself	4.9	4.9	4.9	4.9	4.9	5.0	4.9	4.8
4. in the anamnesis group I was able to practically apply content from my studies	4.1	3.9	4.4	4.3	4.2	4.2	4.1	4.2
5. the participation in the anamnesis group motivated me to actively participate	4.7	4.7	4.8	4.5	4.8	4.7	4.9	4.8
6. I had the feeling that I could contribute my own suggestions at any time	4.6	4.8	4.5	4.7	4.7	4.3	4.8	4.8
7. I learned things in the anamnesis groups that were not yet part of my studies	4.6	4.6	4.8	4.6	4.7	4.8	4.6	4.6
8. questions that arose after the patient interviews were sufficiently discussed	4.6	4.6	4.4	4.6	4.8	4.8	4.8	4.8
9. I received helpful feedback for my patient interview	4.7	4.8	4.6	4.8	5.0	5.0	4.9	4.9

Attachment 3 to: Kunisch R, Zimmermann P, Berges N, Nitschke M, Schweiger F, Seidl M, Weidenbusch M. *E-learning in peer-teaching of patient relations and communication skills at the "Anamnesegruppen" Munich – proof-of-concept and lessons learned.* GMS J Med Educ. 2021;38(1):Doc4. DOI: 10.3205/zma001400

10. the patient interviews were sufficiently debriefed	**4.9**	**4.9**	**4.9**	**4.9**	**5.0**	**5.0**	**4.9**	**4.9**
11. I had the feeling that I could ask questions at any time	4.6	4.7	4.4	4.3	5.0	5.0	4.9	4.9
12. the tutors supported the group constructively in case of problems	4.8	4.9	4.6	4.6	5.0	4.8	5.0	5.0
13. as a result of the anamnesis group I was able to reduce prejudices towards patients	3.9	3.9	3.9	3.9	3.9	3.7	3.9	3.9
14. by participating in the anamnesis group I feel better prepared for future patient contact	4.4	4.4	4.3	4.3	4.6	4.5	4.6	4.8
15. as a result of the participation I was able to reduce my fear towards patients	4	4.1	3.9	4	4.2	3.8	4.3	4.3
16. the participation helped me to better orientate myself regarding my professional future	3.8	3.7	3.9	3.8	3.9	4.3	3.6	3.6
17. as a result of the anamnesis group I learned about my own strengths and weaknesses	4.3	4.3	4.4	4.1	4.5	4.3	4.5	4.4
18. I was able to learn something due to the interdisciplinary nature (e.g. medicine and psychology) of the sessions	4.5	4.4	4.8	4.4	4.6	4.3	4.8	4.9

Attachment 3 to: Kunisch R, Zimmermann P, Berges N, Nitschke M, Schweiger F, Seidl M, Weidenbusch M. *E-learning in peer-teaching of patient relations and communication skills at the "Anamnesegruppen" Munich – proof-of-concept and lessons learned.* GMS J Med Educ. 2021;38(1):Doc4. DOI: 10.3205/zma001400

19. I noticed a difference in motivation between psychology and medical students	**2.8**	**2.9**	**2.8**	**2.6**	**2.7**	**2.2**	**3.0**	**3.0**
20. psychology and medical students were treated differently by the tutors	1.32	1.2	1.5	1.2	1.1	1.0	1.1	1.1
21. I noticed a difference in the quality of patient interviews between psychology and medical students	2.32	2.4	2.3	1.9	1.8	1.7	1.8	1.7
22. I noticed a difference in motivation between elective and non-elective participants	1.1	1.1	1	1.1	1.1	1.0	1.2	1.2
23. elective and non-elective students were treated differently by the tutors	1	1	1	1	1.2	1.3	1.1	1.1
24. I noticed a difference in the quality of patient interviews between elective and non-elective participants	1	1	1	1	1.1	1.0	1.1	1.1
25. I will recommend the anamnesis groups	4.7	4.6	4.9	4.5	4.8	4.8	4.8	4.8
26. I will recommend the anamnesis group as an elective	4.1	4.3	3.9	4.2	4.6	4.0	4.9	4.9
27. I would give the following school grade (1=best; 6=worst) to the anamnesis group:	1.2	1.4	1	1.6	1.1	1.0	1.2	1.1

Total response - summer semester 2019: 21; summer semester 2020: 20

Attachment 3 to: Kunisch R, Zimmermann P, Berges N, Nitschke M, Schweiger F, Seidl M, Weidenbusch M. *E-learning in peer-teaching of patient relations and communication skills at the "Anamnesegruppen" Munich – proof-of-concept and lessons learned.* GMS J Med Educ. 2021;38(1):Doc4. DOI: 10.3205/zma001400
